# Estrogen receptor α interaction of zearalenone and its phase I metabolite α-zearalenol in combination with soy isoflavones in hERα-HeLa-9903 cells

**DOI:** 10.1007/s12550-023-00506-1

**Published:** 2023-10-17

**Authors:** Dino Grgic, Barbara Novak, Elisabeth Varga, Doris Marko

**Affiliations:** 1 Department of Food Chemistry and Toxicology, Faculty of Chemistry, University of Vienna, Währinger Str. 38-40, 1090 Vienna, Austria; 2 University of Vienna, Doctoral School in Chemistry, Währinger Str. 38-42, 1090 Vienna, Austria; 3 dsm-firmenich, ANH R&D center, Technopark 1, 3430 Tulln, Austria; 4 Present address: Unit Food Hygiene and Technology, Institute of Food Safety, Food Technology and Veterinary Public Health, University of Veterinary Medicine, Vienna, Veterinärplatz 1, 1210 Vienna, Austria

**Keywords:** Isoflavones, Mycoestrogens, Combinatory toxicology, Risk assessment, Endocrine disruptors

## Abstract

Risk assessment primarily relies on toxicological data of individual substances, with limited information on combined effects. Recent in vitro experiments using Ishikawa cells, an endometrial carcinoma cell line expressing both estrogen receptor isoforms, demonstrated interactive effects of phyto- and mycoestrogens. The mycoestrogens, zearalenone (ZEN), and α-zearalenol (α-ZEL) exhibited significantly enhanced estrogenic responses in the presence of isoflavones (ISF), depending on substance ratios and concentrations. This study investigated the impact of phyto- and mycoestrogen combinations on estrogenic response following OECD guideline 455, utilizing hERα-HeLa-9903 cells. Test substances included mycoestrogens (ZEN and α-ZEL) and isoflavones (genistein (GEN), daidzein (DAI), and S-equol (EQ), a gut microbial metabolite of DAI). Mycoestrogens were tested in the range of 0.001 to 100 nM, while isoflavones were used at concentrations 1000 times higher based on relevant occurrence ratios. Results showed that ZEN and α-ZEL induced ERα-dependent luciferase expression in concentrations above 1 nM and 0.01 nM, respectively. However, ISF caused a superinduction of the luciferase signal above 1 µM. A superinduction is characterized by an unusually strong or heightened increase in the activity of the luciferase enzyme. This signal is not affected by the estrogen receptor antagonist 4-hydroxytamoxifen (4-OH-TAM), which was additionally used to verify whether the increase of signal is a true reflection of receptor activation. This superinduction was observed in all combinations of ZEN and α-ZEL with ISFs. Contrary to the luciferase activity findings, RT-qPCR experiments and a stability approach revealed lower real ERα activation by ISFs than measured in the ONE-Glo™ luciferase test system. In conclusion, the OECD protocol 455 appears unsuitable for testing ISFs due to their superinduction of luciferase and interactions with the test system, resulting in experimental artifacts. Further studies are necessary to explore structure–activity relationships within polyphenols and clarify the test system’s applicability.

**Supplementary Information:**

The online version contains supplementary material available at 10.1007/s12550-023-00506-1.

## Introduction

The quintessence of toxicology imparts the usage of tools that helps us to understand the harmful effects anthropogenic or natural compounds can have on people, animals, and the environment. Moreover, it comprises the idea of every compound owning the capacity to act adversely, indifferent of their origin, yet depended on the inflicted dosage. New assessments become necessary, as studying the toxicity of single compounds portrays an unrealistic scenario of exposure, hence limiting the predictive powers of such investigations (Bates et al. 2018). So far, risk assessments are still predominantly based on the toxicological data of single substances, which often underestimate the toxicological potential of certain mixtures. Data on combinatorial effects are still scarce, and therefore, in order to ensure extensive health protection and food safety, further studies are required, leading to governmental authorities calling for a need to assess combinatory effects of chemical mixtures (Hartemann and Henstein 2011). Since the inclusion of mathematical methods which dates back to the early 1900s, where Loewe and Munischnek formulated a concept concerning the additivity of chemicals, the assessment of combinatory toxicology gained in popularity (Loewe and Munischnek 1926). Nowadays, newer models such as the Chou and Talalay’s method and the CISNE approach by Garcia-Fuenta et al. have contributed to a further progress of this topic (Chou and Talalay 1983; García-Fuente et al. 2018).

**Fig. 1:**
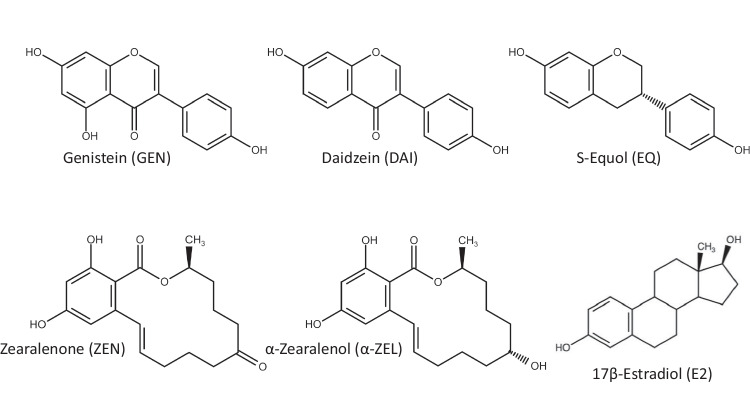
Chemical structures (created with: chemspace.com) of the investigated compounds—the isoflavones genistein (GEN), daidzein (DAI), S-equol (EQ), mycotoxins zearalenone (ZEN), α-zearalenol (α-ZEL), and the hormone 17-β-estradiol (E2)

Recently, interactive effects of phyto- and mycoestrogens were demonstrated in Ishikawa cells using the Chou and Talalay’s method to calculate its combinatory effects (Grgic et al. 2022). Zearalenone (ZEN, Fig. 1) is a well-known mycoestrogen formed by *Fusarium* spp. Upon reductive metabolism, α-zearalenol (α-ZEL) is formed, a step which strongly enhances the estrogenic properties. ZEN is regulated by the European Food Safety Authority (EFSA) for certain animal feed and food stuff, but, for α-ZEL no obligatory limits are applied. However, these fungal metabolites frequently co-occur with non-regulated phytoestrogens as recently summarized for animal feed (Grgic et al. 2021).

In Ishikawa cells, expressing ERα and ERβ, low concentrations of ZEN, α-ZEL and α-zearalanol (0.001–0.01 nM) significantly enhanced the estrogenic response in combination with low concentrations of genistein (GEN), daidzein (DAI) or its gut microbial metabolite S-equol (EQ) (0.001–0.1 µM) compared to their respective single substances (Grgic et al. 2022).

The estrogenic effects of both ISF and ZEN and several of its metabolites are based on the structural and functional similarity to the endogenous hormone 17-β-estradiol (E2). The proposed mechanism of the synergistic estrogenic properties of combinations between ISF and ZEN and its metabolites is based on the different binding affinity of the respective compounds to both isoforms of the estrogen receptor (ERα and ERβ) (Grgic et al. 2022). ISF are well known to preferably bind to the ERβ, whereas ZEN and its metabolites have a high affinity to both isoforms (Nikov et al. 2000; Setchell et al. 2005; Takemura et al. 2007). Therefore, we hypothesize that, in hormone-sensitive cells, synergistic estrogenic effects between phytoestrogens and mycoestrogens in naturally occurring mixtures are mainly due to the presence of both ERs, whereas we do not expect to have synergistic effects when only ERα is present. Our aim in the present study was to demonstrate that synergistic effects of mycoestrogens and ISF are based on the presence of both ER (ERα and ERβ). Using the hERα-HeLa-9903 cells, which solely expresses ERα, synergistic effects were not expected but had to be proven. This cell line was selected according to the Guideline 455 of the Organisation for Economic Co-operation and Development (OECD) (OECD 2015) and the applicability of this guideline for the tested substances is investigated.

## Materials and methods

### Materials

Cell culture flasks and 96-well plates were purchased from Sarstedt (Nürnbrecht, Germany). Cell culture media (Dulbecco’s Minimal Essential Medium (DMEM) and Eagle’s Minimal Essential Medium (EMEM) without phenol red) and supplements (fetal bovine serum (FBS), charcoal–dextran stripped FBS (CD-FBS), blasticidin S HCl and geneticin (G418)) were produced from Gibco and obtained from Thermo Fisher Scientific (Waltham/MA, USA). ZEN, α-ZEL, E2, (Z)-4-hydroxytamoxifen (4-OH-TAM) and sulforhodamine B (SRB) were purchased from Sigma-Aldrich Chemie GmbH (Schnelldorf, Germany). DAI, EQ, and GEN were obtained from Extrasynthese (Genay Cedex, France) whereas dimethly sulfoxide (DMSO), NaCl, KCl, Na_2_HPO_4_, Na_2_HPO_4_ * 2 H_2_O, and KH_2_PO_4_ were purchased from Carl Roth GmbH + Co. KG (Karlsruhe, Germany). The CellTiter-Blue^®^ Cell Viability Assay Kit and ONE-Glo™ EX luciferase assay system were obtained from Promega Corporation (Madison/WI, USA).

### Cell line

The modified human cervical cell line for the identification of ERα agonists and antagonists also known as “hERα-HeLa-9903” was purchased from Sigma-Aldrich Chemie GmbH (Schnelldorf, Germany). Cell stocks were stored in liquid nitrogen containers and 2 weeks prior to the start of the in vitro experiments, cells were taken in culture. They were cultivated in an incubator at 37 °C with 5% CO_2_ and 95% humidity using the growth medium consisting of DMEM, supplemented with 5% (*v/v*) heat-inactivated FBS, 16 µg/mL blasticidin S HCl and 800 µg/mL G418. Cells of the passage number 5 were split at a confluency of about 80% and kept in culture up to the maximum passage number of 40. Before the assays were performed, the growth medium was aspired and replaced with the assay medium consisting of EMEM supplemented with 5% CD-FBS.

### ONE-Glo™ EX luciferase (OGL) assay

The assays were performed in 96-well plates following the instructions of the manufacturer (Promega 2007). Per well, 10,000 hERα-HeLa-9903 cells in assay medium were seeded and grown for 3 h. Thereafter, the assay medium was replaced with the incubation solutions consisting of various concentrations of ZEN, α-ZEL, GEN, DAI, and EQ as single substances or in the respective combinations in assay medium. The substances were previously dissolved in DMSO in 2000 times higher concentrations than the tested concentration, followed by dilution in the assay medium. In case of single substance testing and for the solvent control, DMSO was added to reach 0.1% in the final incubation solutions. Concentrations ranged from 0.001 to 100 nM in case of ZEN and its metabolites and 0.001 to 100 µM in case of ISF, with 1:10 dilution steps in between. E2 (1 nM) served as positive control and 4-OH-TAM (10 μM) as a negative control. All experiments were performed in technical triplicates and at least five independent biological replicates.

Following the 24-h incubation, the supernatants were discarded and 100 µL of a ONE-Glo™ EX luciferase incubation solution (1:1 dilution of OGL reagent and EMEM (5% CD-FBS)) was added and shaken for 5 min. Subsequently, 90 µL of the supernatant of each well was transferred to a new, white 96-well plate. The luminescence of the white plate was directly measured with a gain of 140 using the Victor V3 1240 Multilabel Counter from Perkin Elmer (Waltham/MA, USA) or the Cytation 3 Cell Imaging Multi-Mode Reader from Biotek^®^ (Winooski/VT, USA). Final results were referred to the solvent control (0.1% in DMSO) in percentage.

### OGL assay stability approach

Cell seeding was performed as described in the "ONE-Glo™ EX luciferase (OGL) assay" section. Subsequently, cells were incubated with 10 nM ZEN for 24 h. Following the incubation, the supernatant was removed and 100 µL of the OGL incubation solution was added and shaken for 5 min. Subsequently, 90 µL of the supernatant of each well was transferred to a new, white 96-well plate and right before measuring the bioluminescence, 10 µL of different concentrations (0.01–100 µM) of the ISF (GEN, DAI, EQ) were added to investigate whether the signal intensity is increasing over a 3-h period. The luminescence of the white plate was measured with a gain of 140 as stated above.

### Coupled CellTiter-Blue^®^ and SRB cytotoxicity assay

The incubation solutions were prepared as described for the OGL assay (see respective section) and the same single substances, combinations thereof and solvent control (0.1%) were used. After the 3-h seeding period of the cells, the plates were incubated with the compound of interest for 24 h. All experiments were performed at least in five independent biological replicates with technical triplicates each. Following the incubation period, the subsequent steps were carried out in the dark: The supernatant was aspirated and 100 µL of a 1:10 CellTiter-Blue^®^ (CTB) and EMEM (5% CD-FBS) solution were added and incubated for 50 min. Thereafter, 80 µL of each well was transferred into a black microwell plate and fluorescence was measured using an excitation wavelength of 560 nm and an emission wavelength of 590 nm with a gain of 65 with the Victor V3 1240 Multilabel Counter from Perkin Elmer (Waltham/MA, USA) or the Cytation 3 Cell Imaging Multi-Mode Reader from Biotek^®^ (Winooski/VT, USA). Final results were referred to the solvent control (0.1% DMSO) in percentage.

The SRB assay was conducted immediately after the CTB assay. Following the removal of the remaining CTB solution the cells in each well were fixed with 10 µL of a 50% trichloroacetic acid solution in distilled water. The 96-well plates were placed for 1 h into the refrigerator at 4 °C. Upon this cold incubation, the plates were carefully rinsed twice with tap water and then left to dry in a dark surrounding. Once the plates had dried, 50 µL of the SRB solution was pipetted into each well. The dye was left to stain the proteins for 1 h, again in a dark setting at room temperature. Afterwards the dye was rinsed off twice with tap water and twice with a 1% acetic acid solution. Here great care was taken to avoid the removal of the stained proteins from the bottom of the wells and the well plates were left to dry in the dark. Once the plates had dried, the SRB solution bound to the protein components of the cells was dissolved in 100 µL of an alkaline Tris base solution (0.30 g tris(hydroxymethyl-) aminomethane solved in 250 mL distilled water) by shaking for 5 min in the plate reader (Victor V3 1240 Multilabel Counter from Perkin Elmer (Waltham/MA, USA) or the Cytation 3 Cell Imaging Multi-Mode Reader from Biotek^®^ (Winooski/VT Vermont, USA)). Subsequently, the absorbance was measured at 570 nm and as for the CTB; the final results were referred to the solvent control (0.1% DMSO) in percentage.

### Quantitative real-time PCR (RT-qPCR)

For the RT-qPCR, cells (hERα-HeLa-9903) were seeded in assay medium in 12-well plates at a density of 150,000 cells and allowed to grow for 24 h. Incubation solutions contained 0.1% DMSO and the test compounds and compound mixtures at various concentrations. After the same incubation time as the OGL-assay (24 h), cells were washed and subsequently the total RNA was extracted following the RNeasy Mini Kit protocol from Qiagen. The following reverse transcription was performed according to the QuantiTect-Reverse-Transcription manual (Qiagen 2009) to transcribe 1 µg RNA to cDNA. Then, RT-qPCR was performed using primers for the firefly luciferase encoding gene with the following oligonucleotide sequences for reverse primer 5′-GCCTCACCTACCTCCTTGCT-3′ and forward primer 5′-CTTCGTGACTTCCCATTTGC-3′, as well as the endogenous control primers delta-aminolevulinate synthase (1HS_ALAS1_1_SG, QT00073122), actin beta (Hs_ACTB_1_SG, QT00095431) and glyceraldehyde-3-phosphate dehydrogenase (Hs_GAPDH_1_SG, QT00079247) with SYBR green as fluorescent probe. The amplification the following setting was applied: 95 °C for 2 min, followed by 40 repetition cycles of: 95 °C (15 s), 55 °C (15 s), 72 °C (60 s). Thereafter, a melting curve analysis using the following parameters was performed: 15 s at 95 °C, 1 min at 60 °C, in 0.5 °C steps to 94 °C for 15 s. All samples were normalized to the mean of the endogenous control genes, in the case of GEN and combinations thereof (β-actin and GAPDH) and for DAI and EQ and mixtures thereof (ALAS1 and GAPDH). Quantification was performed according to Livak and Schmittgen (2001) using the 2-ΔΔCt method resulting in the depiction of fold-changes in comparison to the solvent control. We tested the transcriptional activity of GEN, DAI and EQ (0.1, 1 µM), and ZEN (10 nM) and combinations of ISF (1 µM) and ZEN (10 nM).

### Statistics

In order to generate substantial data, the measurements of OGL assay and cytotoxicity concerning the various combinations of ISF, ZEN, and ZEN metabolites were conducted in technical triplicates in addition to a minimum of five independent biological replicates. RT-qPCR experiments were performed in technical duplicates and in at least four independent biological replicates (*n* ≥ 4). Then, the mean values for the biological replicates were calculated upon eliminating outliers based on the method of Nalimov and verifying normality following Shapiro-Wilk.

Further statistical analysis was performed using the software Origin Pro^®^ 2021 (OriginLab Corporation, Northampton/MA, USA), with significance levels of 5%, 1%, and 0.1%, respectively (#, x = *p* < 0.05; ##, xx = *p* < 0.01; ###, xxx = *p* < 0.001). Significant differences were evaluated via one-way analysis of variance (ANOVA) followed by Fisher’s least significant difference (LSD) post hoc test. Cytotoxicity and RT-qPCR results were evaluated by using one-way Student’s *t*-test.

## Results

### OGL assay single substances

**Fig. 2:**
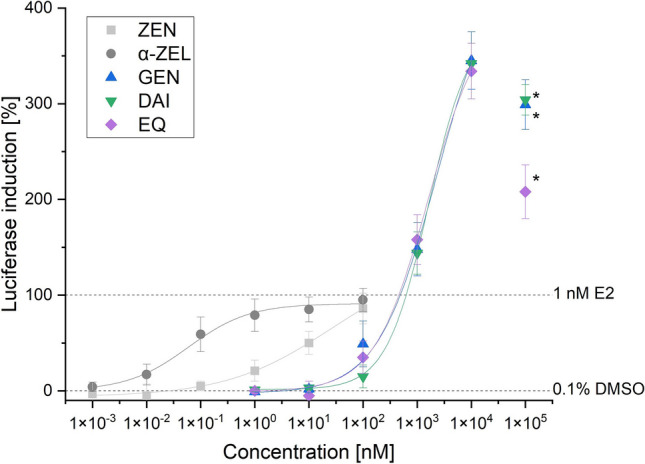
Sigmoidal-dose-response curve fits (created with: Origin) of the estrogen-dependent activation of luciferase induction in hERα HeLa 9903 cells caused by ZEN, α-ZEL and the ISF GEN, DAI and EQ after 24-h incubation. Results are depicted as mean ± standard deviation of at least 7 biological replicates (measurements with different cell passages), calculated from the mean value of three technical replicates (repeated measurements with the same cell passage on the same plate). Outliers after the Nalimov outlier test as well as values marked with asterisk (*) were not included in the sigmoidal-dose-response curve fit. Effects of the solvent control (0.1% DMSO) and 1 nM E2 as positive control were set to 0 and 100%, respectively

The induction of an estrogenic response for both mycoestrogens ZEN and α-ZEL was approximately the same. A concentration dependent increase in signal was observed and a concentration of 100 nM showed the highest luciferase induction of 95% (α-ZEL) and 86% (ZEN), related to 1 nM E2 (Fig. 2). Furthermore, in a concentration dependent manner the tested ISF induced an increase in luciferase activity up to a concentration of 10 µM with the highest induction by GEN, followed by DAI and EQ, with 345%, 342%, and 334%, respectively (Fig. 2). At a concentration of 100 µM, a decrease in luciferase induction was observed for all three ISF.

**Fig. 3:**
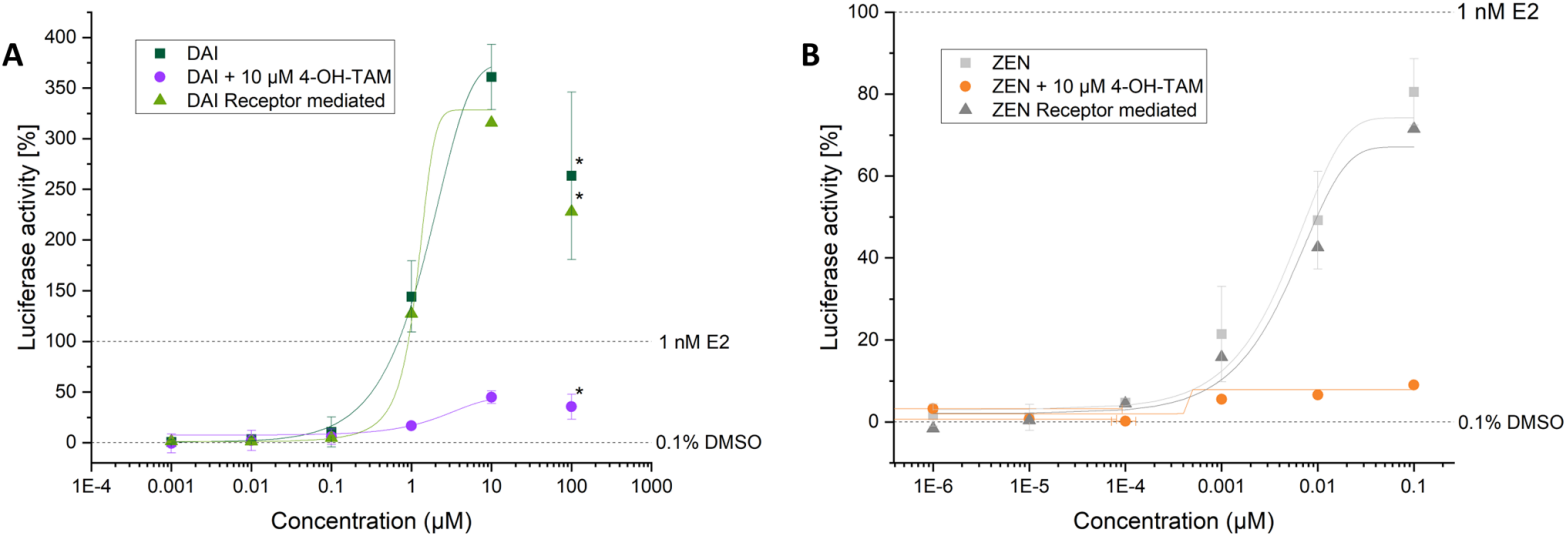
Total and receptor mediated (ERα) dose-response curves of DAI and ZEN (created with: Origin). **A** The dose-response curves of DAI in a concentration range between 0.001 µM and 100 µM are shown. In dark green the total luciferase activity induced by DAI is depicted while the red dots show the luciferase activity of DAI + 10 µM 4-OH-TAM (estrogen receptor α antagonist). In light green the calculated (total luciferase activity − the activity induced by DAI + 10 µM 4-OH-TAM) receptor mediated luciferase activity is shown. **B** The dose-response curves of ZEN in a concentration range between 1E^−6^ and 0.1 µM (hence a factor 1000 lower as tested for DAI). In light gray the total luciferase activity induced by ZEN is depicted. The orange dots show the luciferase activity of ZEN + 10 µM 4-OH-TAM and in dark gray the calculated (total luciferase activity − the activity induced by ZEN + 10 µM 4-OH-TAM) receptor mediated luciferase activity is provided. Outliers after the Nalimov outlier test as well as values marked with asterisk (*) were not included in the sigmoidal-dose-response curve fit. Effects of the solvent control (0.1% DMSO) and 1 nM E2 as positive control were set to 0 and 100%, respectively

Exemplarily for combinations of DAI, additional assays with the ER antagonist 4-OH-TAM (10 µM) were conducted, according to the OECD protocol 455 for unambiguous confirmation of ER-mediated estrogenic activity (OECD 2015). 4-OH-TAM inhibits the activity of the ERs and by using it as an antagonist; it can be assessed whether the observed effects of a test substance are indeed mediated by the estrogen receptor. If the effects are reduced in the presence of 4-OH-TAM, it suggests that the test substance is likely interacting with the estrogen receptor. This helps in differentiating between estrogenic effects mediated through the estrogen receptor and other non-specific effects that might occur due to different mechanisms. 4-OH-TAM significantly lowered these apparent estrogenic activities, although at higher concentrations of ISF (≥ 1 µM), nonspecific interactions were also observed (Fig. 3). The true estrogenic activity that is mediated through ERα is calculated as the differences between 4-OH-TAM untreated and treated samples. Nevertheless, the higher transcriptional activity of DAI (≥ 1 µM) compared to E2 remains even after calculating the difference between 4-OH-TAM treated and untreated samples.

By calculating sigmoidal dose-response curve fitting the effective concentration that induces 50% response (EC_50_) can be determined. The obtained EC_50_ values for the mycoestrogens were 0.057 ± 0.004 nM (α-ZEL) and 24.4 ± 0.4 nM (ZEN), and for the ISF 2.3 µM ± 0.4 (GEN), 1.6 ± 0.2 µM (EQ), and 1.6 ± 0.7 µM (DAI).

### OGL assay of combinations

**Fig. 4:**
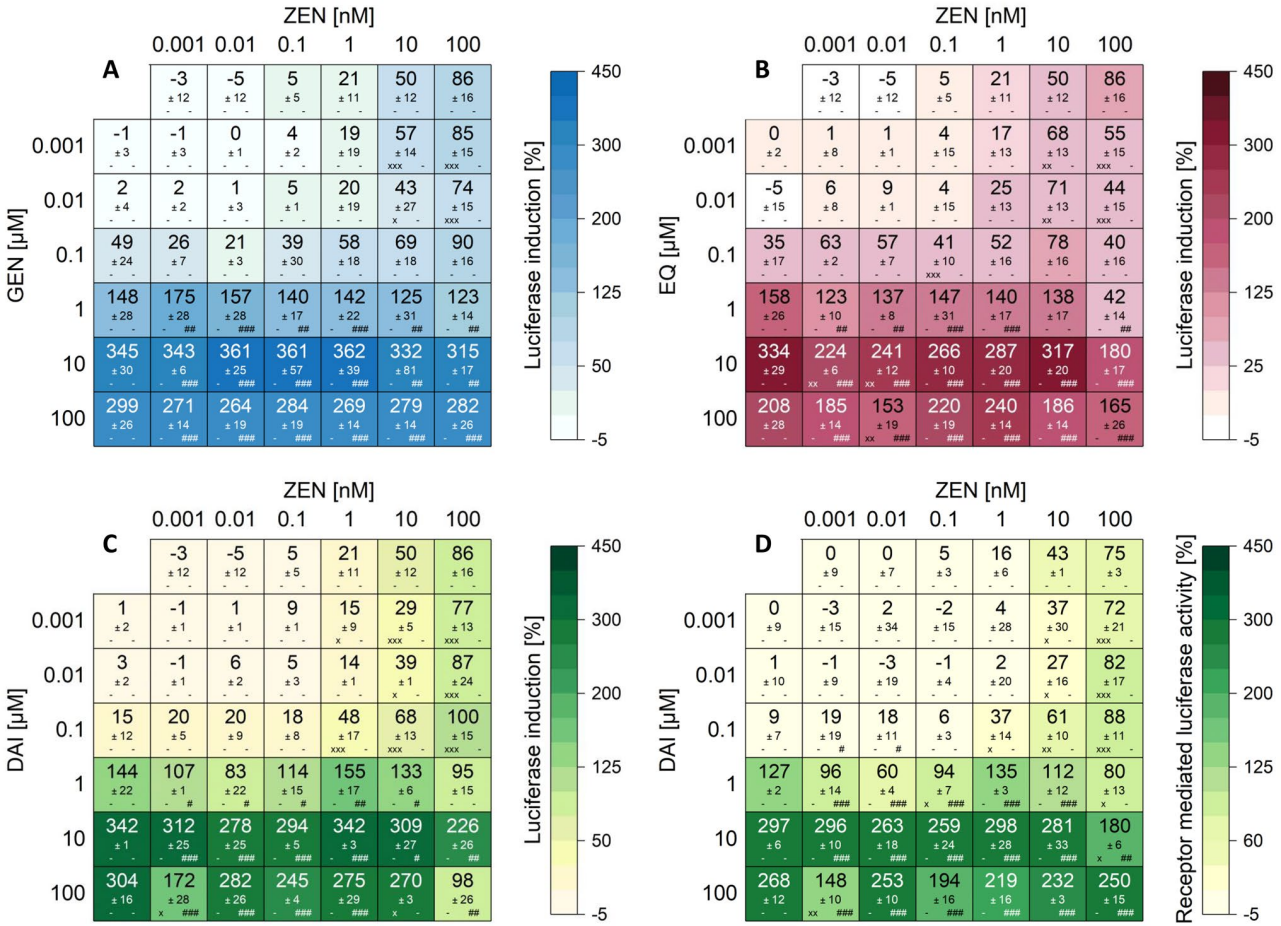
Effects of the combination of ISF with ZEN on the luciferase induction after 24 h of incubation using the hERα HeLa 9903 cell line (created with: Origin). Heatmaps indicating effects of single substances and combinations of GEN (**A**), EQ (**B**), DAI (**C**) and receptor mediated luciferase induction of DAI (**D**) with ZEN on the luciferase induction in hERα HeLa 9903 cells after 24 h incubation. Results are depicted as mean ± standard deviation of at least five biological replicates. Outliers after Nalimov outlier test were excluded. Effects of the solvent control (0.1% DMSO) and 1 nM E2 as positive control were set to 0 and 100%, respectively. The color code indicates the strength of the effects. Normal distribution of data was tested according to Shapiro-Wilk normality test and significance by one-way ANOVA. Significant differences of effects to the respective single substance concentration were indicated with x = p < 0.05, xx = p < 0.01 and xxx = p < 0.001 in case of isoflavones and # = p < 0.05, ## = p < 0.01 and ### = p < 0.001 in case of ZEN. “-” corresponds to no significant difference to the respective concentration of the single substance

**Fig. 5:**
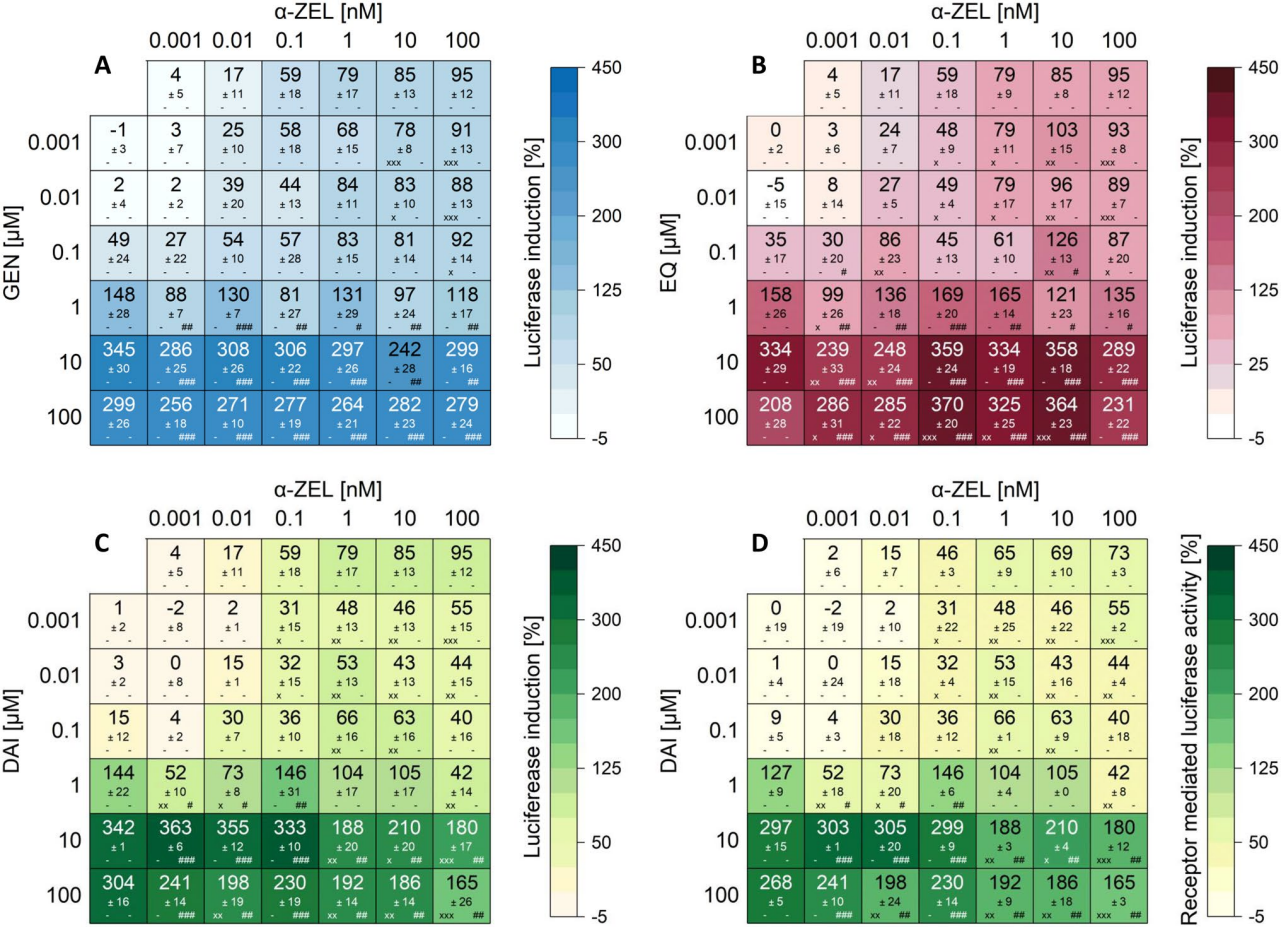
Effects of the combination of ISF with α-ZEL on the luciferase induction after 24 h of incubation using the hERα HeLa 9903 cell line (created with: Origin). Heatmaps indicating effects of single substances and combinations of GEN (**A**), EQ (**B**), and DAI (**C**) and receptor mediated luciferase induction of DAI (**D**) with α-ZEL on the luciferase induction in hERα HeLa 9903 cells after 24-h incubation. Results are depicted as mean ± standard deviation of at least five biological replicates. Outliers after Nalimov outlier test were excluded. Effects of the solvent control (0.1% DMSO) and 1 nM E2 as positive control were set to 0 and 100%, respectively. The color code indicates the strength of the effects. Normal distribution of data was tested according to Shapiro-Wilk normality test and significance by one-way ANOVA. Significant differences of effects to the respective single substance concentration were indicated with x = *p* < 0.05, xx = *p* < 0.01, and xxx = *p* < 0.001 in case of the isoflavones and # = *p* < 0.05, ## = *p* < 0.01, and ### = *p* < 0.001 in case of α-ZEL. The symbol “-” corresponds to no significant difference to the respective concentration of the single substance

All measured effects of single substances and combinations are compiled in the heatmaps (Figs. 4 and 5). Results are expressed as percentage of induction, where 0 and 100% represent the values of the solvent control (0.1% DMSO) and 1 nM E2, respectively. Significant luciferase activation of combinations compared to their respective single substances are indicated by “x” in the case of ISF and by “#” for mycoestrogens. The color code of these heatmaps indicates the strength of the effect, which enables a visual interpretation of results of all tested combinations.

For all data sets, binary mixtures of ISF with ZEN and α-ZEL showed similar trends. Overall, no significant increase of luciferase activity was observed for all combinations and concentrations compared to their respective single substances (Figs. 4 and 5). Only a few exceptions were able to potentiate the luciferase activity significantly (100 µM EQ + different α-ZEL concentrations) (Fig. 5B). ISF concentrations ≥ 1 µM alone and in combination with mycoestrogens surpassed the luciferase activation of 1 nM E2 (values above 100%) (Figs. 4 and 5). However, binary mixtures suppressed the luciferase activity compared to the respective single substances. Starting at a concentration of 0.001 µM for ISF, the addition of ZEN or α-ZEL (0.001–100 nM) lowered the induction of luciferase activity. This effect was more pronounced at higher ISF concentrations (1–100 µM). A concentration dependent increase in luciferase activity was observed for both mycoestrogens, whereas for all tested ISF, a decrease in luciferase activity was seen at the highest concentration of 100 µM. Furthermore, in combinations of 100 µM ISF together with ZEN or α-ZEL (0.001–100 nM) a decrease in luciferase activity was noticed.

### OGL assay stability approach

**Fig. 6:**
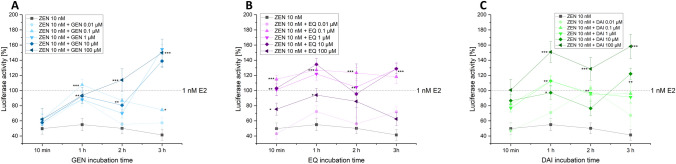
Effects of ISF on luciferase stability: Luciferase activity of ZEN alone and with different concentrations of GEN (**A**), EQ (**B**), and DAI (**C**) in hERα HeLa 9903 cells. The cells were incubated for 24 h with ZEN and the Isoflavones were added right before measuring the bioluminescence over a period of 3 h. Results are depicted as mean ± standard deviation of at least five biological replicates. Outliers after Nalimov outlier test were excluded. Effects of the solvent control (0.1% DMSO) and 1 nM E2 as positive control were set to 0 and 100%, respectively. Significant differences of effects to the respective single substance concentration were indicated with *p < 0.05, **p < 0.01, and ***p < 0.001. Please notice that the luciferase activity is only displayed between 40 and 200%. (created with: Origin)

In the OGL stability approach, cells were incubated with 10 nM ZEN for 24 h and thereafter for 5 min with the OGL incubation solution. Thereafter, different ISF concentrations were added to the cell free supernatant. As seen in Fig. 6, ISF increased the stability of the enzyme which led to higher activities over time compared to the luciferase activity induced by ZEN alone. This was observed immediately after adding the ISF and over a time period of 3 h.

### Cytotoxicity (CTB and SRB)

The metabolic activity and the protein content were assessed using the CTB and SRB assay, respectively, to determine the cytotoxicity of single compounds and combinations of phyto- and mycoestrogens. The purpose of the inclusion of cytotoxic measurements was to rule out possible artifacts induced by the compounds due to cell death or proliferation.

#### Single substances

**Fig. 7:**
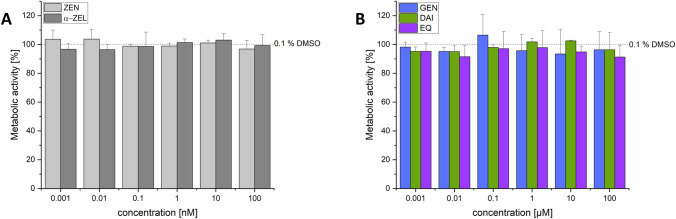
Effects of the single substances on the metabolic activity (%) measured by CTB after 24-h incubation in hERα HeLa 9903 cells for ZEN and α-ZEL (**A**) and GEN, DAI and EQ (**B**). Values were referred to the solvent control (0.1% DMSO) as 100%. Results are depicted as mean + standard deviation of at least eight biological replicates, calculated from the mean value of three technical replicates. Outliers after Nalimov outlier test were excluded. Significant differences of effects between the solvent control and the incubation solutions were calculated by one-sample Student’s t-test (*p* < 0.05), but no significant difference was observed. (created with: Origin)

As single substances neither ZEN nor α-ZEL at all applied concentrations induced a reduction in metabolic activity in the utilized test system (Fig. 7A). Furthermore, neither the cell protein amount (SRB test system) was affected by these mycoestrogens in the same concentration range (Supplements Fig. 1A).

For all three tested ISF no significant difference in metabolic activity was detected (Fig. 7B). Additionally, the protein content was not affected when compared to the solvent control (0.1% DMSO) (Supplements Fig. 1B).

#### Cytotoxicity of combinations

**Fig. 8:**
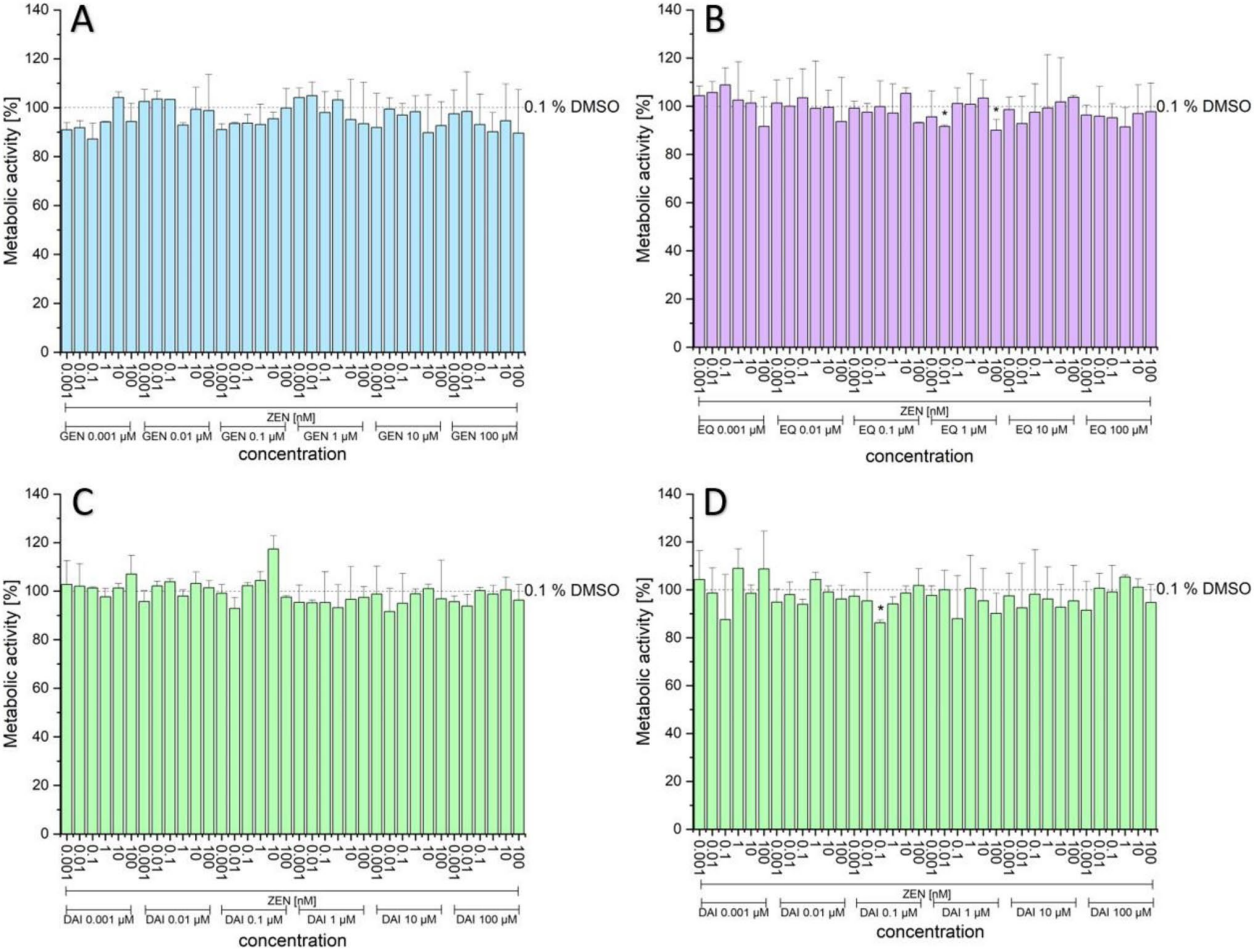
Effects of the combination of ZEN with ISF on the metabolic activity (%) measured by CTB of different combinations and concentrations after 24-h incubation in hERα HeLa 9903 cells. GEN (**A**), EQ (**B**), DAI (**C**), and DAI + 10 µM 4-OH-TAM (**D**) with ZEN (nM). Values were referred to the solvent control (0.1% DMSO) as 100%. Results are depicted as mean + standard deviation of at least four biological replicates, calculated from the mean value of three technical replicates. Outliers after Nalimov outlier test were excluded. Significant differences of effects between the solvent control and the incubation solutions were calculated by one-sample Student’s t-test. Significances are indicated with asterisk (*) (*p* < 0.05). (created with: Origin)

**Fig. 9:**
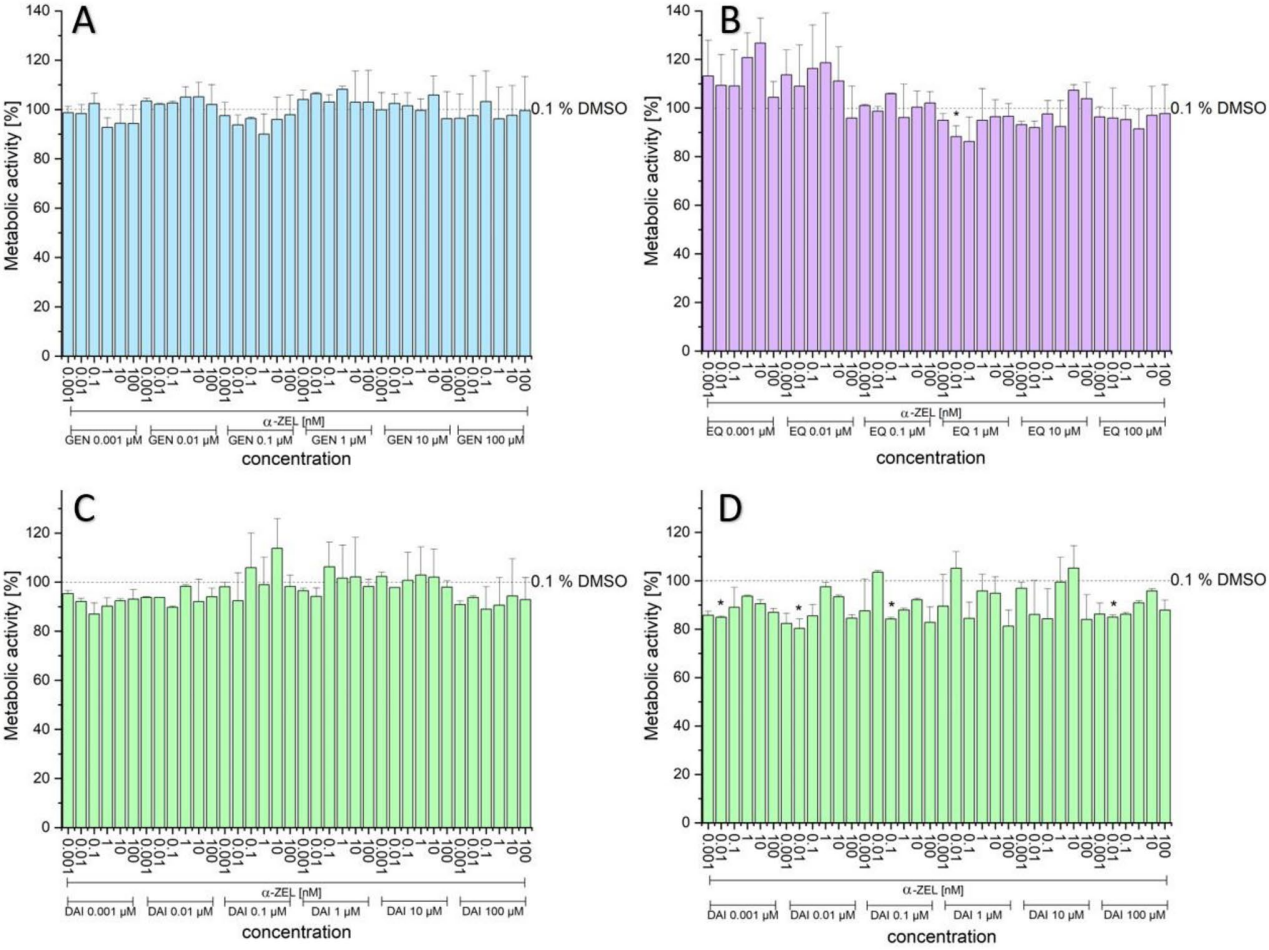
Effects of the combination of α-ZEL with ISF on the metabolic activity (%) measured by CTB of different combinations and concentrations after 24-h incubation in hERα HeLa 9903 cells. GEN (**A**), EQ (**B**), DAI (**C**), and DAI + 10 µM 4-OH-TAM (**D**) with α-ZEL (nM). Values were referred to the solvent control (0.1% DMSO) as 100%. Results are depicted as mean + standard deviation of at least four biological replicates, calculated from the mean value of three technical replicates. Outliers after Nalimov outlier test were excluded. Significant differences of effects between the solvent control and the incubation solutions were calculated by one-sample Student’s t-test. Significances are indicated with asterisk (*) (*p* < 0.05). (created with: Origin)

As shown in Figs. 8 and 9, no significant decrease in metabolic activity for most of the tested combinations of binary mixtures of the tested phyto- and mycoestrogens was detected. However, for some combinations an increased tendency in the two applied systems was observed (Fig. 9B). Furthermore, the combinations of DAI with 10 µM 4-OH-TAM showed a decrease in number of cells compared to the cells which were untreated with 10 µM 4-OH-TAM, albeit just a few significant differences were detected (Fig. 9D). Moreover, the cell protein amount using the SRB assay, showed for most combinations no significant differences compared to the solvent control. Cells treated with 10 µM 4-OH-TAM showed a tendency to decrease the protein content; however, for most combinations, this trend was not significant.

### RT-qPCR

**Fig. 10:**
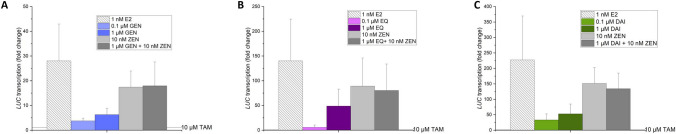
Effects of ISF and ZEN and combinations thereof on gene transcription (created with: Origin). Alterations in gene transcription of hERα HeLa 9903 cells incubated for 24 h with 1 nM E2, 10 µM 4-OH-TAM, 0.1 and 1 µM of GEN, EQ and DAI, 10 nM ZEN and combinations of 1 µM GEN, EQ and DAI with 10 nM ZEN. Results are depicted as mean ± standard deviation of at least four biological replicates. Outliers after Nalimov outlier test were excluded. For the RT-qPCR experiments with GEN, β-actin and GAPDH were used as housekeeping genes, whereas for DAI and EQ, GAPDH and ALAS1 were applied. As a result, different fold changes of the luciferase encoding gene were observed when comparing the experiments

In the applied concentrations, ISF only increased the transcriptional activity slightly and were not able to surpass the induction of luciferase mRNA induced by 1 nM E2 (Fig. 10). ZEN induced luciferase mRNA levels significantly and was comparable to the induction of luciferase using the OGL assay. The combination of 1 µM ISF and 10 nM ZEN showed the same increase in luciferase mRNA levels as ZEN alone. For the RT-qPCR experiments with GEN, β-actin and GAPDH were used as housekeeping genes, whereas for DAI and EQ, GAPDH, and ALAS1 were applied. As a result, different fold changes of the luciferase encoding gene were observed when comparing the experiments.

## Discussion

As previously reported, it was demonstrated that binary mixtures of ISF together with ZEN and several of its metabolites have synergistic estrogenic effects using the Ishikawa cell model, which expresses both isoforms of the ER (Grgic et al. 2022). It is hypothesized that these enhanced estrogenic effects are based on the presence of both ER isoforms α and β. While ZEN and its metabolites have a relative high affinity to bind to both ERs, ISF preferably interact with ERβ (Nikov et al. 2000; Setchell et al. 2005; Takemura et al. 2007). In the present study, we tested selected combinations using the hERα-HeLa-9903 cell model expressing solely the ERα. According to an OECD protocol, this assay should be used for detecting estrogen receptor agonists and antagonists [13]. Based on the previous results our hypothesis was that no enhanced estrogenic effects should be observed in the hERα-HeLa-9903 cell line due to the lack of ERβ.

Performing the OGL assay, α-ZEL in a concentration range between 0.01 and 10 nM had a higher potency to induce luciferase compared to ZEN. It is well known that the estrogenic effect of the phase I metabolite is more pronounced compared to its parent compound (Molina-Molina et al. 2014; Vejdovszky et al. 2017; Mendez-Catala et al. 2020; Grgic et al. 2022). At a concentration of 100 nM, both mycoestrogens had a similar impact on luciferase induction of 95% (α-ZEL) and 86% (ZEN). Interestingly, when comparing the estrogenic effect of these two mycoestrogens between different cell lines, 10 times higher concentrations were required in hERα-HeLa-9903 cells to induce the same estrogenic effects when compared to Ishikawa cells (Grgic et al. 2022). As for the ISF a superinduction of luciferase is observed, no direct comparison to other cell lines is possible. This superinduction of luciferase by ISF in hERα-HeLa-9903 cells was already described by Gramec Skledar et al. and was in accordance with the results of these experiments (Gramec Skledar et al. 2020).

When examining the luciferase response triggered by mycoestrogens and ISF, it was observed that ZEN and α-ZEL initiated an increase in luciferase activity at concentrations of 0.1 and 0.01 nM, respectively. In contrast, ISF demonstrated a noticeable signal increase starting at a concentration of 100 nM. This divergence in concentration thresholds suggests the higher binding affinity of ISF to the estrogen receptor alpha (ERα), underscoring the superior estrogenic potency of mycoestrogens compared to ISF. It is worth noting, however, that while ZEN and α-ZEL exhibited substantial luciferase induction, they did not surpass the induction levels achieved by E2, a known estrogenic compound. Conversely, ISF showed the ability to exceed the activities of E2. This, however, does not fully reflect receptor-mediated interactions and is known as the phenomena superinduction.

As expected, combinations of mycoestrogens and ISF did not show increased luciferase activity compared to the induction of their respective single substances (Figs. 4 and 5). However, ISF was found to induce luciferase activities up to three times higher compared to the positive control of 1 nM E2.

Therefore, the next step was to verify whether or not the increased firefly luciferase activity is a true reflection of increased transcriptional activity. Thus, the mRNA expression of the firefly luciferase gene was measured in ERα-HeLa-9903 cells. We tested the transcriptional activity of GEN, DAI and EQ (0.1, 1 µM), and ZEN (10 nM) and combinations of ISF (1 µM) and ZEN (10 nM) (Fig. 10). Those concentrations were chosen based on the results of the OGL assay aiming for a concentration without (0.1 µM) and with (1 µM) superinduction in luciferase. This allowed the assessment of the validity of the OGL-experiment for ISF. In the applied concentrations, ISF only slightly increased the transcriptional activity and were not able to surpass the induction of luciferase mRNA induced by 1 nM E2. This indicates that the superinduction observed by the ISF in the ERα-HeLa-9903 cells is not mediated by the receptor activation and therefore, not a true reflection of its binding affinity to the ERα in this system. ZEN was able to induce luciferase mRNA levels significantly and was comparable to the induction of luciferase in ERα-HeLa-9903 cells. The combination of 1 µM GEN and 10 nM ZEN showed the same increase in luciferase mRNA levels as ZEN alone. This observation would support the theory that for synergistic estrogenic effects both estrogen receptors are required, with different binding affinities of two substances to the ER α and β.

It is known that there might be a direct interaction of ISF with the reporter enzyme leading to stabilization (decreased degradation) of the enzyme. It has been proposed that certain compounds directly bind to, and stabilize, the firefly luciferase reporter enzyme thereby increasing its half-life (Pfitscher et al. 2008). The interaction between the bioactive chemicals and the luciferase results in inhibition of the enzyme activity at the same time resulting in stabilization of the enzyme (Thomas 2008). These luciferase-stabilizing compounds are referred to in the literature as luciferase inhibitors.

It can be expected that upon rupture of the cells the inhibitor will dissociate leading to increased activity of the luciferase enzyme due to this stabilizing effect. Thus, ISF at concentrations above 1 µM may interact with luciferase thereby stabilizing the enzyme so that upon lysis of the cells and dissociation of ISF from the enzyme due to dilution, increased activity can be measured as compared to the situation without an added stabilizer/inhibitor (Sotoca et al. 2010). It has been recently shown, that a series of bioactive compounds, that inhibit and thereby stabilize the firefly luciferase enzyme will result in an increased luminescence signal (Pfitscher et al. 2008).

Therefore, in a further approach, we performed a luciferase reporter assay to determine if the ISF are able to increase the stability and protect against degradation. We incubated the cells for 24 h with 10 nM ZEN. Subsequently, right before measuring the bioluminescence, different concentrations (0.01–100 µM) of ISF were added to investigate whether the signal intensity is increasing over a 3-h period. As depicted in Fig. 6, the addition of different concentrations of ISF to 10 nM ZEN increased the stability of the enzyme which led to higher activities over time compared to the luciferase activity that was induced by ZEN alone. The addition of ISF independent of the applied concentration led to increased signal intensity. This was observed immediately after adding the ISF and increase over this relative short incubation time. This observation supports our hypothesis that ISF increase the stability of luciferase and therefore such high bioluminescence signals can be induced by ISF as seen in the experiments of combinations between ISF and ZEN.

To conclude, in our experiments we aimed to display the importance of the presence of both isoforms of the ER, ERα, and ERβ to induce synergistic estrogenic effects upon co-occurrence of myco- and phytoestrogens. Indeed, in cell lines bearing only one isoform of the ER, as it was the case for the hERα HeLa 9903, no enhancement in estrogenicity was observed up to a concentration of 0.1 µM of the ISFs. In higher ISF concentrations, we elucidated that assays according to the OECD guideline 455 are not a true reflection of receptor activation and are therefore not applicable for interaction studies with these types of compounds. When applying the OGL assay, it is important to test for any inferences resulting in the non-applicability of the test system.

### Supplementary Information

Below is the link to the electronic supplementary material.

Supplementary file1 (PDF 457 KB)
