# Supplementary material for: Estrogen receptor α interaction of zearalenone and its phase I metabolite α-zearalenol in combination with soy isoflavones in hERα-HeLa-9903 cells
Source: Mycotoxin Res. 2023 Oct 17;40(1):97–109. doi: 10.1007/s12550-023-00506-1 (PMC10834624; doi:10.1007/s12550-023-00506-1)
Supplement: Supplementary file 1 — Supplementary file1 (PDF 457 KB) [file 12550_2023_506_MOESM1_ESM.pdf]

Supplementary material:

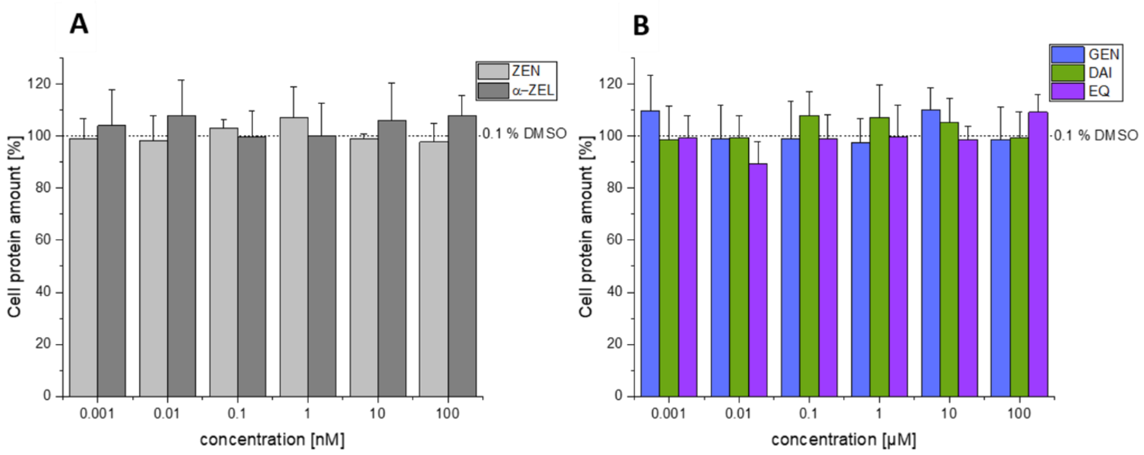

Supplements Figure 1: Effects of single substances on the cell protein amount (created with: Origin)

Impact on the cell viability [%] measured by sulforhodamine B (SRB) of different concentrations after 24 h incubation in hERα HeLa 9903 cells. Values were referred to the solvent control (0.1 % DMSO) as 100 %. Results are depicted as mean + standard deviation of at least eight biological replicates, calculated from the mean value of three technical replicates. Outliers after Nalimov outlier test were excluded. Significant differences of effects between the solvent control and the incubation solutions were calculated by one-sample Student's *t*-test ($p < 0.05$), but none were observed.

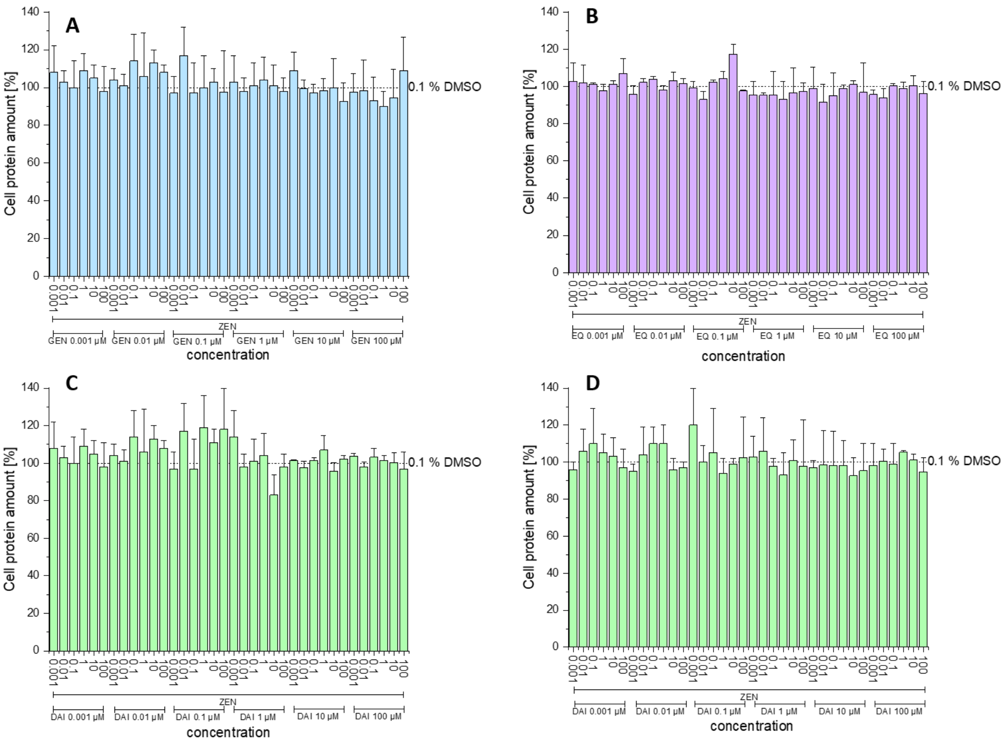

Supplements Figure 2: Effects of the combination of zearalenone (ZEN) with isoflavone on the cell protein amount (created with: Origin)

Impact on the cell viability [%] measured by sulforhodamine B (SRB) of different combinations and concentrations after 24 h incubation in hERα HeLa 9903 cells. Genistein (GEN) (A), S-equol (EQ) (B), daidzein (DAI) (C) and DAI + 10 µM 4-OH-tamoxifen (D) with ZEN. Values were referred to the solvent control (0.1 % DMSO) as 100 %. Results are depicted as mean + standard deviation of at least four biological replicates, calculated from the mean value of three technical replicates. Outliers after Nalimov outlier test were excluded. Significant differences of effects between the solvent control and the incubation solutions were calculated by one-sample Student's *t*-test ($p < 0.05$), but none were observed.

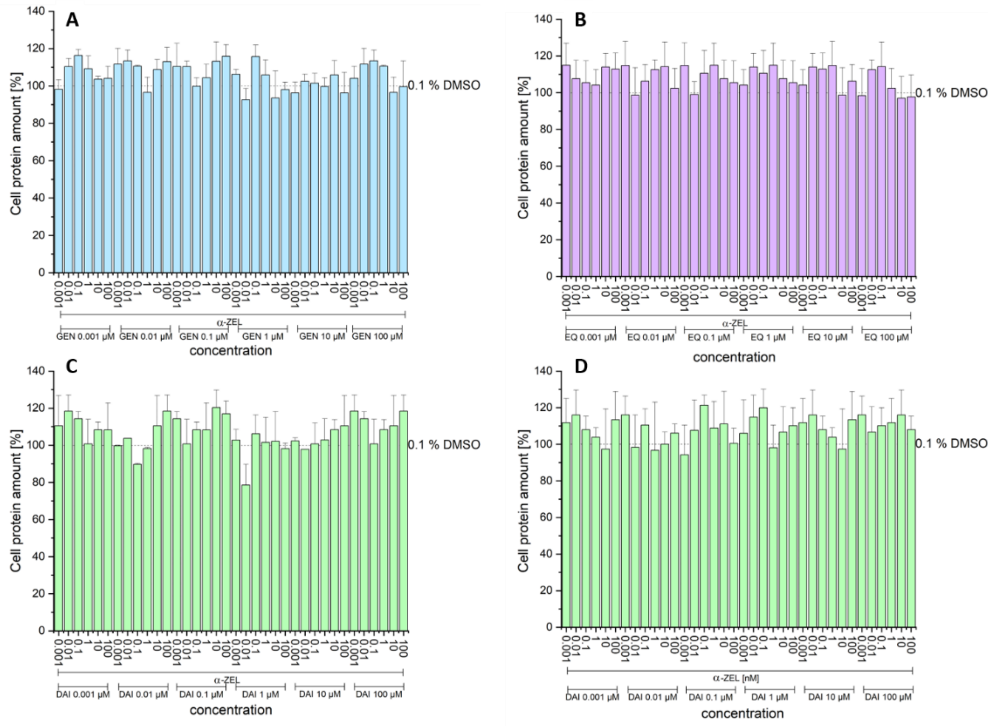

Supplements Figure 3: Effects of the combination of α-zearalenol (α-ZEL) with isoflavone on the cell protein amount (created with: Origin)

Impact on the cell viability [%] measured by sulforhodamine B (SRB) of different combinations and concentrations after 24 h incubation in hERα HeLa 9903 cells. GEN (A), EQ (B), DAI (C) and DAI + 10 µM 4-OH-tamoxifen (D) with α-ZEL. Values were referred to the solvent control (0.1 % DMSO) as 100 %. Results are depicted as mean + standard deviation of at least four biological replicates, calculated from the mean value of three technical replicates. Outliers after Nalimov outlier test were excluded. Significant differences of effects between the solvent control and the incubation solutions were calculated by one-sample Student's *t*-test ($p < 0.05$), but none were observed.
